# Children’s Attentional Processes in Outdoor and Indoor Environments: The Role of Physiological Self-Regulation

**DOI:** 10.3390/ijerph192013141

**Published:** 2022-10-12

**Authors:** Lucia Mason, Benedetta Zagni, Francesca Bacchin, Carlotta Frison, Sara Scrimin

**Affiliations:** Department of Educational Psychology and Socialisation, University of Padova, 35122 Padova, Italy

**Keywords:** attention, green environments, indoor environments, nature, children, physiological self-regulation, emotion, heart rate variability

## Abstract

Previous research has documented that exposure to green spaces has the beneficial effects of attention restoration and stress reduction. This study investigated the effects of indoor (classroom) and outdoor (green school garden) environments on attentional processes in interaction with emotion and physiological self-regulation. Children in third and fourth grades (*n* = 42) completed a school-related emotional Stroop task assessing the effects of outdoor and indoor classroom backgrounds when facing positive and negative stimuli. Children’s attentional patterns in a task completed in both environments were also assessed. Heart rate variability was registered at rest as an index of physiological self-regulation. The results revealed that children were less distracted from negative emotional materials when presented with outdoor compared with indoor background stimuli. Greater selective attention and sustained attention were shown in the green than in the classroom environment. Moreover, sustained attention varied in relation to physiological self-regulation but only when performing the task indoor.

## 1. Introduction

In recent years, there has been an increasing interest in the positive effects that natural environments may have on children’s cognition, especially attention, wellbeing, psychological health, and pro-sociality, also when considering school contexts [1,2,3,4]. Systematic reviews have also documented the various benefits of nature across age levels [3,5]. Importantly, such benefits are even associated with passive relationships with greenness, that is, when the natural environment is not used or incorporated in an activity [5]. These reviews, however, only addressed long-term children’s exposures to nature. We are also interested in short-term exposures to greenness during a school day as instructional and learning activities can take place not only indoor but also in green areas [6,7]. To extend current knowledge, the aim of our study was to investigate the role of the two types of environments—indoor classroom and green outdoor space—in school-age children’s attentional performance. Comparing indoor and outdoor environments has theoretical relevance to understand the contextual factors that better support academic functioning, as well as practical relevance to implement interventions aimed at improving school achievement. In the next sections we will focus on attention and physiological self-regulation in relation to such environments to ground our study on the main issues of the relevant literature and to provide the rationale for the investigation. 

### 1.1. Attention and Exposure to Natural Environments 

Attention is an essential function in school activities as students must be able to direct their attentional focus on specific stimuli, such as the teacher’s verbal explanation. When selected attention is maintained on a specific stimulus over a long period of time to a high degree, it is considered sustained attention or concentration [8]. Attention is associated with academic achievement [9,10]. Indeed, attention is a fundamental cognitive resource that is involved in executive functions and self-regulation tasks [11], which in turn contribute to academic achievement [12]. However, attentional resource is limited in amount and subject to depletion as maintaining it for long times leads to fatigue. Thus, attention should be restored to allow subsequent successful cognitive performance. According to the Attentional Restoration Theory (ART), attention is renewed even after a short exposure to green space [13]. Kaplan based his theory on a distinction between two types of attention: involuntary attention and voluntary or directed (sustained) attention. Involuntary attention is automatic attention that does not require mental resources when stimuli attract our attentional focus. Voluntary or directed attention is not automatically invoked since it requires our effort to focus, for instance, on an uninteresting stimulus, and to remain focused on it. Directed attention keeps distractions under control through the use of inhibition and is susceptible to fatigue. Natural environments are rich of stimuli (e.g., bird songs) that softly attract our involuntary attention minimizing voluntary attention with irrelevant, distracting stimuli being ignored more readily through the mechanism of inhibitory control of distractions. As a consequence, the capacity-limited attentional system depletes more slowly in the greenness [11,13]. Multiple studies reported that voluntary attention is restored in students, e.g., [14] after short-term contact with greenness during a school day as documented by a very recent systematic review [15]. Outdoor green environments, such as green school yards and playgrounds, are also perceived by children as more restorative than outdoor built environments [16,17].

To sum up, the cognitive benefit of exposure to nature reflects on attentional capacity. However, attention is also strictly related to affect as evidenced in neuroscientific studies [18]. Control of attention and emotion are therefore two important aspects of cognitive functioning to be considered when investigating the positive effects of natural environments. 

### 1.2. Attention and Emotion: The Importance of Physiological Self-Regulation

Sensory processing and attention are influenced by stimulus relevance and such relevance is determined by the preexisting motivational state [19]. It means that motivationally relevant stimuli capture individuals’ attention as they are the most salient for their current needs. This is particularly relevant within the school where both the classroom physical and socio-emotional environments can become more salient than an academic task and hence potentially distract students by capturing their attention. That is, students’ attention may focus on environmental stimuli with emotional content rather than on a cognitive task as the former better respond to students’ current affective needs. 

To investigate the impact of emotional material on attentional performance a widely used experimental paradigm in cognitive psychology is the emotional Stroop task, e.g., [20]. The emotional Stroop interference effect is represented by longer response time in the requested task (i.e., naming the ink color of a word or a frame) when the stimulus has an emotional valence (i.e., negative emotional words vs. neutral words), meaning that individuals’ attention is captured by the negative emotional content and distracted from the main task. In primary-school children, research has documented an attentional bias toward school-related stressors, specifically words that described school threats. Young adolescents with low school wellbeing and high negative emotionality were more inclined to biased attention for school threats than those with low negative emotionality [21]. 

Indeed, individual differences may moderate the effects of emotionally negative materials. One of these is physiological self-regulation as indexed by heart rate variability (HRV). It reflects the variation in the time intervals between heartbeats, which is a function of the parasympathetic branch of the autonomic nervous system [22]. HRV is an important index of the individual’s ability to adapt and respond to environmental demands [23]. Greater resting HRV is considered to represent better mental and physical wellbeing as a relatively stable trait that may play a role in how a person responds in everyday life to a given situation [24]. Specifically, HRV reflects the autonomic modulation of emotional responding to environmental challenges [25]), which mirrors the activity of the prefrontal cortex according to the Neurovisceral Integration Model [26]. 

Empirical evidence indicated that resting HRV is linked with attention control when a person is presented with emotional stimuli. Individuals with higher HRV are more able to resist distractors and maintain attention [27]. Studies with children have demonstrated that their physiological regulation positively relates to effortful control that includes both attentional focusing and shifting [28]. To our knowledge, only one study has investigated the role of HRV in an emotional Stroop task in the school context, and the focus was on classroom climate [29]. The study provided evidence that HRV is a physiological correlate of students’ self-regulation in response to environmental demands. However, students’ physiological self-regulation has not been investigated in relation to attentional performance in different environments, such as an indoor classroom or an outdoor green school garden. With this regard, there is a need to bring together issues from three separate lines of research regarding attention, emotion, and physiological self-regulation when considering the environments where students’ performance takes place. 

### 1.3. Research Questions and Hypotheses

To fill in this gap, therefore, and extend current research on the benefits of short exposure to nature, we considered a novel and worthwhile approach for investigating the role that indoor and outdoor school environments may have in the interplay between the core concepts of attention, emotion, and physiological self-regulation. The indoor environment is a typical classroom, while the outdoor environment is a green, natural space. As reported in the previous sections, the outdoor green environment has been proven to be beneficial for attentional performance as exposure to greenness captures involuntary attention, so the capacity-limited voluntary attentional system depletes more slowly, in accordance with the ART theory [13]. In the present work we examined attentional patterns (reaction times and emotional interference index) in a computerized school-related emotional Stroop task (step 1) and attentional patterns (selective attention and sustained attention) in a paper-and-pencil task (step 2). Specifically, in the first step, through a typical lab task we investigated children’s allocation of attentional resources in response to different emotional school-related stimuli (positive vs. negative) embedded in different environmental backgrounds (outdoor green setting or indoor classroom setting). In the second step, children were assessed in real outdoor and indoor school environments (school garden vs. classroom) through a typical task of selective and sustained attention. Thus, in each step of our research work we examined the role of the environment—either depicted in the graphical stimuli or the real environment where the task was executed—on children’s attentional performance. In each step we also examined the moderating role of physiological self-regulation in the relationship between indoor/outdoor environment and attention. Thus, the following logically related research questions (RQ) guided the work.

- Step 1: Emotional Stroop Task

RQ1: Do children’s allocation of attentional resources as indexed by reaction times in an emotional Stroop task differ in relation to the stimulus valence and the environmental context in which they are situated? Based on the aforementioned literature on attention bias and negative emotionality, we hypothesized that response times would be longer for emotionally negative than positive stimuli as the former can tax attentional and processing resources more than the last ones [21,29]). The difference in response times (and thus in the amount of allocated attention) between the positive and negative stimuli is considered as the emotional interference index. That is, when children spend more resources in looking at the negative than positive stimuli and take more time to respond to the color frames, emotional interference is higher.

RQ2: Does the emotional interference index change as a function of the environmental context? If this difference emerges, is it moderated by children’s physiological self-regulation as reflected in their HRV? Based on the above-reviewed literature, we hypothesized higher emotional interference when children would be presented with stimuli having an indoor classroom background compared to an outdoor green environment [17]. Moreover, an interactive effect of the environmental context and HRV would emerge. Even if, to our knowledge, these variables have not yet been considered simultaneously in the same investigation, it is reasonable to expect children with higher heart rate variability at rest to be better self-regulated while facing more taxing classroom environments and be less distracted by them, as well as to perform better on the attention task.

- Step 2: Attentional Task

RQ3: Do children’s selective and sustained attention scores differ in relation to the environment in which the attention task is executed? Based on evidence of the benefits of exposure to greenness for attentional performance, we hypothesized greater selective attention and greater sustained attention when children would be in the green environment as it depletes their limited attentional resources more slowly and distracts them less by irrelevant stimuli compared to the indoor environment of the classroom [3]).

RQ4: Does their HRV moderate this relationship? Based on evidence on the interaction between visual environment, attention, and physiological self-regulation, we hypothesized that HRV would moderate sustained attention performance. This would particularly benefit children with lower HRV only in the classroom as its visual environment requires more attentional control over distracting stimuli than the green environment that captures involuntary attention [3,24].

## 2. Methods 

### 2.1. Study Design

The study investigates the role of greenness on students’ attention also assessing the possible moderating role of physiological self-regulation. Students were assessed multiple times with a multimethodological within-subject approach that included physiological indexes, behavioral measures, and paper and pencil tasks (see Figure S1 in Supplementary Materials). Specifically, first children were individually assessed to register (a) heart rate variability at rest as a trait-like index of physiological regulation. This allowed to have an accurate registration of each child ability to regulate and adapt to the environment, an important individual characteristic that could moderate the effect of the environment on attentional tasks. In the same individual session children’s (b) reaction times were recorded in a modified version of the emotional Stroop task (Step 1). This task allowed to study the effect of an indoor vs. outdoor background on an attentional task. Here, we were interested in studying whether the emotional interference effect would be attenuated by a greenly background. Last, children (c) as a class were collectively assessed on a paper and pencil attention task twice, that is, inside the classroom and outside in the greenness (Step 2). In this way we examined the effects of direct exposure to the two environments on the attentional task.

### 2.2. Participants

A total of 42 children in the third (*n* = 19) and fourth 4th (*n* = 23) grades (females = 19; 47.5% *M*_age_ = 9.67, *SD* = 0.66) participated in the study. They did not have any certified disability or learning and behavioral problems. Parental written permission and children’s verbal assent were required for participation; in addition, written informed consent was obtained from school principal. The study was approved by the Ethics Committee of the pertinent institution.

### 2.3. Measures

#### 2.3.1. Emotional Stroop Task

**Figure 1:**
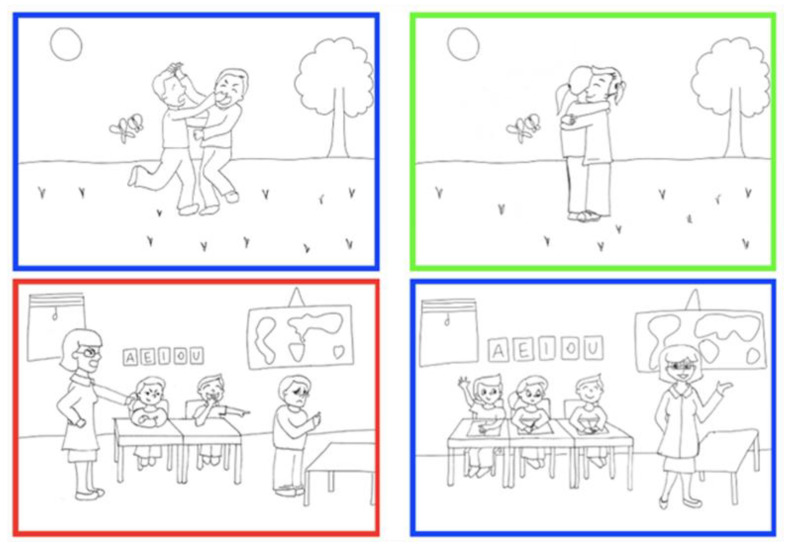
Examples of Positive and Negative, Indoor and Outdoor Matched Stimuli from the Emotional Stroop Task.

It was a slightly modified version of the task used in Scrimin et al.’s study [29] (p. 153), which consisted of four negative and four positive black-white scenes of school-related social interactions. For the purpose of this study, the eight original scenes (4 positive and 4 negative) were doubled changing the background of the image. Eight stimuli had an indoor typical classroom environment in the background: four negative (i.e., two typical peer interactions that may evoke negative emotions and two teacher-student negative interactions) and four positive scenes which differed only for the emotional valence as characters, backgrounds, and degrees of social contact were exactly the same. The negative scenes are such as they represent two peer interactions and two teacher-student interactions that may elicit negative emotions. Examples of the former are the case of two children who are fighting each other or the case of three children who are bullying another child. Examples of negative scenes of teacher-student interactions are that of a teacher who is questioning a student in front of the class or a teacher who is yelling at a student. The positive scenes represented social interactions that may elicit positive emotions, for instance two children who are hugging or a teacher who is praising a student. For the current study, however, to the emotional valence variable we added the environmental variable, that is, the scenes were included in a usual indoor context or in an outdoor context. A set of eight more stimuli were created for the present study using the exact same scenes but changing the background to an outdoor environment. The environmental elements where matched in terms of number and dimensions: whereas in the original classroom environment background elements were tables, chairs, and a blackboard, in the outdoor background elements were grass, flowers, a three, and the sun. Each of the 16 black-and-white pictures was presented with each of the three possible color frames (i.e., blue, green, and red), resulting in a total of 48 stimuli. Examples of visual stimuli are shown in Figure 1.

Pictures were presented on an Asus laptop with a 17-inch screen through OpenSesame software (Portland, OR, USA), version 3.3.4 [30] in a randomized order. For each trial, a fixation cross at the center of the screen appeared for 750 ms, followed by a picture with a colored frame which remained until the child gave a response. Students were asked to name the frame color as fast as possible while disregarding the content of the picture. Before the individual administration of the task, instructions were presented on the screen and read by the experimenter who ensured their comprehension. Children also completed four practice trials before starting the task. Reaction times were registered using a voice-activated microphone in front of the child at a distance of 12 cm, which was connected to the computer. Emotional interference index was computed by subtracting averaged responses times to positive pictures from responses times to negative pictures. Positive scores indicated longer responses time when negative pictures were presented, whereas negative scores indicated shorter response times to negative than positive pictures.

#### 2.3.2. Attentional Task 

As a measure of selective and sustained attention, we used the Bell test [31] as in the study by Amicone et al. [32] who involved students of the same age. It is a paper-and-pencil, nonverbal standardized test that consists of four printed sheets, each one containing one-dimensional scattered figures of several objects (e.g., trees, fishes) including 35 black bells. Participants are asked to find as many bells as they can in a fixed time for each sheet which has more than a hundred small pictures (including the 35 bells) scattered randomly around the white page. This reduces the possible practice effect that could be present in other attention tests where stimuli are presented on a matrix. A score of selective attention is given on the basis of the total number of bells marked by the child on the first sheet. A score of sustained attention is given by the total number of bells marked by the participant on the fourth sheet. Incorrectly marked stimuli are not computed in the final score (maximum score for each sheet = 35). Children completed a trial sheet to ensure their comprehension of task instructions before starting it. The task was performed in a counterbalanced order both indoor in the classroom environment and outdoor in the school garden. 

#### 2.3.3. Physiological Self-Regulation 

Heart rate was registered by means a POLAR sensor that was positioned on the child’s chest using a monitoring device that encodes biological signals (ProComp Infiniti, Thought Technology, Montreal, QC, Canada). The electrocardiogram (ECG) signal was recorded continuously for 8 min via a 12-bit analog-to-digital converter with a sampling rate of 256 Hz and stored sequentially for analysis. The raw ECG data was then processed through Kubios-HRV 2.2 (Kuopio, Finland) software in order to assess the rate of each heartbeat and derive the series of normal inter-beat intervals (IBIs), computed as the difference in ms between successive R-waves. In addition, in order to detect and correct artifacts, the raw signal was visually inspected, and a piecewise cubic splines interpolation method was performed when necessary. Then, we computed the square root of the mean squared differences (rMSSD) of successive IBIs. rMSSD is an index of short-term heart period fluctuations and is thought to reflects vagally mediated influence of the parasympathetic activity on the sinoatrial node [22].

### 2.4. Procedure

In order to establish a friendly relationship with the participants, researchers joined the classroom and interacted in advance with children weekly. In fact, the present study was conducted thanks to a long-term collaboration between the University and the school. The researchers who collected the data are considered part of the school staff, as such it was possible for them to organize activities that took place both in the classroom and in the greenness (i.e., large school garden with grass and big trees). During the first assessment, children were tested individually during the morning in a quiet room of the school. Once the child entered the room, the POLAR sensor was placed on the child’s thorax and the child was invited to sit and relax while watching a calming video during which the ECG was recorded. After eight minutes the sensors were removed. At this point the emotional Stroop task was performed. Each child was instructed to play a game on a laptop computer which consisted in naming as fast as possible the correct color of the frame of the picture appearing on the screen. After completing the task, students had a short talk with the researcher sharing their opinion on the session before returning to their class. The second session took place the following week. The same researchers joined the class in a regular academic activity for two hours. During the two-hour time, the text of a story was read by the teacher and then children were asked to answer a number of text comprehension questions. At the end of the lesson the Bells test was administered. Of note is that this session took place two times within two weeks with the same students (within-subjects research design) in the same weekday and at the same time of the school day, but one time in the classroom and the other time in the nature, in a counterbalanced order. Half children executed the task indoor for the first time, the other half executed it outdoor (and vice versa) to ensure that familiarity with the task would not interfere with the environment where the task was executed. It should also be noted that the attentional task was selected in order not to cause a significant practice effect in the second repetition (see Measures section). Teachers and researchers were also the same in indoor and outdoor environments.

### 2.5. Analytical Plan

Statistical analyses were conducted using R software [33], version 3.4.4. First, data were graphically examined for skewness, kurtosis, and outliers. The Kolmogorov–Smirnov test confirmed normalcy (all ps > 0.05) for all variables; hence, no transformations were necessary. Row physiological data were processed by mean of Kubios software to detect and correct artifacts. Specifically, the raw signal was visually inspected together with the tachogram and a piecewise cubic splines interpolation method was performed when necessary, in order to remove artifacts and clean data. Then, we computed the square root of the mean squared differences (rMSSD) of successive inter beat intervals (IBIs) by means of the Kubios software. The rationale for choosing this specific time domain index lies in its reliability in being a good correlate of parasympathetic vagal activity. 

In the Step 1 data from the modified version of the emotional Stroop task were considered. Initially, reaction time data were edited for each child to remove error trials in the emotional Stroop (the error rate was lower than 0.7%) as well as any trials more than two standard deviations from the mean, as these trials likely represent lack of attention to the task. Mean reaction times were then calculated for each of the four categories of emotional stimuli (i.e., positive and negative, with outdoor and classroom background). 

Subsequently, to answer RQ1 about differences in reaction times in response to stimuli with different valence (positive vs. negative) and background environment (outdoor vs. classroom) a 2 × 2 univariate ANOVA was performed while controlling for age. As a next step the emotional interference index was calculated by subtracting the response times (and thus in the amount of allocated attention) in response to the negative stimuli from the time of response to the positive stimuli. This difference indicates greater amount of time spent by children (i.e., resources spent) looking at the negative compared with the positive stimuli that in turn makes them take more time to respond to the color frames. Longer times imply greater emotional interference.

The interference index was used to answer RQ2. By means of a univariate ANOVA while controlling for age we analyzed if the emotional interference index changed as a function of the environmental context. Furthermore, a linear regression model allowed us to test the direct and interactive effects of environmental background and physiological self-regulation as heart rate variability (HRV) on children’s emotional interference. The model included the emotional interference index as the dependent variable and environmental background (outdoor vs. classroom), HRV, and their interaction as predictors. As before, age was controlled. 

In Step 2, to answer RQ3 we performed a multivariate analysis of variance to compare children’s selective attention and sustained attention scores when completing the task in the green environment (school garden) and in the classroom, while controlling for age. Last, two linear regression analyses were performed to answer RQ4. The first model had students’ selective attention as a dependent variable while the second had sustained attention. The models included direct and interactive effects of the environment in which the task was performed (outdoor vs. classroom) and HRV, while controlling for age. Significant interactions were explored by means of a slope analysis.

## 3. Results

### 3.1. Step 1: Emotional Stroop Task 

#### 3.1.1. Allocation of Attentional Resources and Environmental Background (RQ1)

A significant difference was found between positive and negative stimuli, *F*(1, 1242) = 5.43, *p* = 0.01, *d* = 0.10, with longer reaction times in response to negative (*M* = 760.04, *SD* = 292.96) than positive stimuli (*M* = 733.81, *SD* = 258.55). However, reactions times in response to environmental background did not statistically differ, *F*(1, 1242) = 2.52, *p* = 0.11, *d* = 0.09, even if reaction times in response to outdoor green backgrounds were shorter (*M* = 701.06, *SD* = 245.30) than reactions time in response to classroom backgrounds (*M* = 722.60, *SD* = 262.99). The statistically non-significant interaction between the two conditions was removed from the model for the sake of simplicity.

#### 3.1.2. Emotional Interference, Physiological Self-Regulation, and Environmental Background (RQ2) 

A significant difference emerged for the emotional interference index in response to stimuli with different background environments (outdoor vs. classroom), *F*(1, 40) = 2.44, *p* = 0.02, *d* = 0.17. Specifically, emotional interference was smaller in response to outdoor (*M* = 15.21, *SD* = 53.81) than classroom backgrounds (*M* = 38.42, *SD* = 55.71), indicating that children were less distracted by emotionally negative stimuli when presented in an outdoor environment. 

Subsequently, the model resulting from the linear regression analysis with the emotional interference index as the dependent variable, explained 12% of the variance. As can be seen in Table 1, environmental background (outdoor vs. classroom) was significantly associated with emotional interference, with higher interference in response to stimuli with a classroom than outdoor background. 

In addition, a direct negative link with HRV as well as a two-way interaction between environmental background (outdoor vs. classroom) and HRV emerged. However, follow-up simple slope analysis indicated that none of the slopes were significant (see Figure S2 in Supplementary Material).

### 3.2. Step 2: Attention in Real Green and Classroom Environments

#### 3.2.1. Selective and Sustained Attention (RQ3)

Findings revealed a significant difference in the selective attention scores across the two environments (outdoor vs. classroom), *F*(1, 40) = 13.93, *p* = 0.001, *d* = 1.02. Specifically, children had grater selective attention scores, that is correctly identified the bells in the test, when they were in the outdoor green environment (*M* = 19.19, *SD* = 4.53) than in the classroom (*M* = 14.70, *SD* = 4.27). Similarly, performance in sustained attention was significantly greater, *F*(1, 40) = 7.49, *p* = 0.001, *d* = 0.06, when completing the task outdoor (*M* = 33.71; *SD* = 1.46) than in the classroom (*M* = 32.57, *SD* = 2.41).

#### 3.2.2. Attention and Physiological Self-Regulation (RQ4)

As shown in Table 2, the first model with selective attention as the dependent variable revealed a significant effect of the environment with children scoring higher in selective attention when performing outside in the school garden compared to inside the classroom. No other significant association was found. 

**Figure 2:**
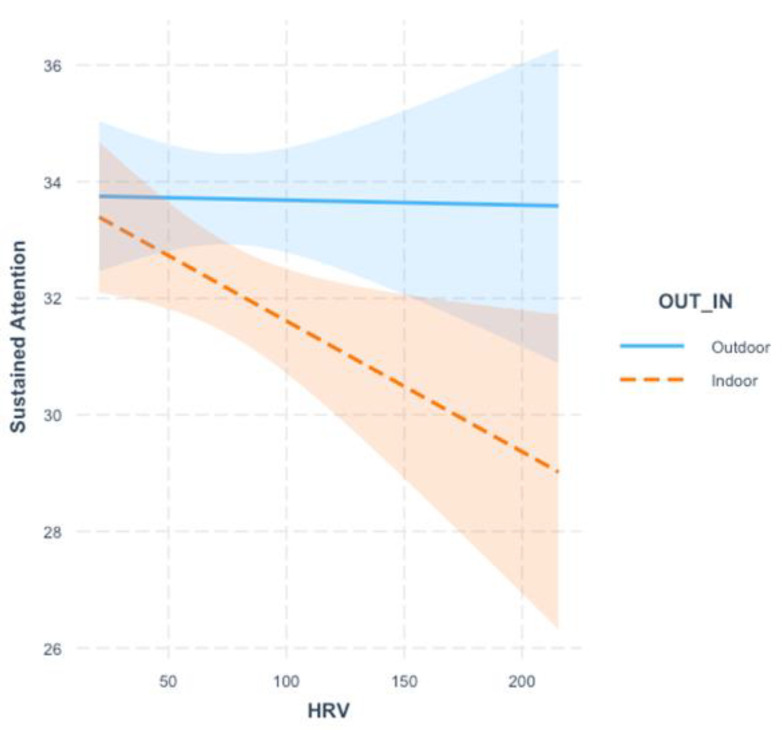
Simple Slopes for the Interaction Between Environment (Outdoor vs. Classroom) and Heart Rate Variability (HRV) on Children’s Sustained Attention.

The second model with sustained attention as the dependent variable, revealed a significant main effect of the environment with children showing better sustained attention when performing outside in the school garden than inside the classroom. Importantly, a significant interaction was also found. As depicted in Figure 2, follow-up simple slope analysis indicated that when performing in the classroom, children with higher HRV displayed significant less sustained attention (*B* = −0.02, *SE* = 0.01, *t* = −2.43, *p* = 0.02) than children with lower HRV. In contrast, no effect of HRV was observed among children performing in the outdoor green environment (*B* = −0.01, *SE* = 0.01, *t* = −0.09, *p* = 0.93).

## 4. Discussion

This study investigated attention in school-age children, comparing their performance in outdoor green environments and regular indoor classroom environments while also assessing the role of physiological self-regulation. As the first step we investigated attentional patterns in terms of reaction times to a school-related emotional Stroop task. As expected, children responded with significant more selective attention when exposed to positive compared to negative stimuli, hence confirming previous studies reporting that images representing negative social interactions require greater attentional resources than images depicting positive ones [29,34]. In terms of reaction times, in the Stroop task the background stimuli did not have a significant effect. It may probably due to the fact that within the stimulus, the emotional component expressed by the characters was more relevant than the environmental background. Indeed, studies investigating visual processing of pictorial stimuli through eye-tacking methodology have shown that early fixations were more stimulus-driven and a center bias was also found [35]. 

Our participants’ attention was captured by the emotional scene presented in the center. Yet, when computing the emotional interference index, a significant effect of the background was found. Children’s attention was less captured by negative social interactions occurring at school when these took place in an outdoor green environment compared to an indoor classroom. That is, the distractive effect of negative emotional school-related stimuli is less present when the background has distinguishing marks such as trees, grass, and flowers compared to blackboard, desks, and chairs. These data show how, even in a computer-based lab task, negative emotional cues are better dealt with when inserted in an outdoor environment. This outcome is in line with research that provides accumulated evidence of the positive effects of outdoor green settings on emotional wellbeing and health in children [1,2,4]. In this regard, it has also been demonstrated that even a school window with a green view can reduce stress in high-school students as well as sustain their attentional capacity compared to a window with a built view [36]. 

Interestingly, these data seem to show that the characteristics of an outdoor background help students to perform better on an attentional task despite the presence of emotionally distracting conditions in a way that is similar to high physiological self-regulation (HRV). As a matter of fact, confirming findings of previous investigations [29], the current study reveals a direct negative effect of HRV on the emotional interference index. Children with greater capacity to adapt to the environment and self-regulate are less distracted from the task by negative emotional cues. It should be noted that, in line with the Neurovisceral Integration Model [26], HRV is a physiological correlate of inhibitory control abilities which are core skills for adopting appropriate behaviors in the classroom. At school students’ inhibition of attention towards irrelevant stimuli is linked with the ability to focus on the requested task. Inhibitory control is also closely interrelated with emotional self-regulation and heart rate variability [37] which, in our study, are linked to attention allocation in the emotional Stroop task with school-related stimuli. In sum, the first step of the study underlines the importance of outdoor stimuli and self-regulatory abilities when dealing with emotionally disturbing events at school while being asked to perform a simple basic attentional task. 

In the second step of our work we investigated attention performance using a task in real outdoor and indoor environments. Children were assessed outside in the school garden and inside in the classroom. In line with our expectations, they performed better in terms of both selective and sustained attention when completing the task in the greenness. These outcomes are aligned with previous studies indicating that in green environments attentional resources deplete more slowly as nature helps maintaining directed attention, preventing distractions from interfering with the purposeful activity [38,39]. 

In addition, sustained attention was also moderated by physiological self-regulation as indexed by HRV. Specifically, overall, students’ sustained attention outdoor was always fairly high. However, when inside the classroom, children with lower HRV performed better than those with high HRV. This finding may seem counterintuitive at a first glance as higher HRV has been documented as positively associated with cognitive performance [27]). It should be noted that low HRV at rest indicates an overall poor ability to adapt to changes in the environment and it has been found to be linked with poor cognitive flexibility [23]. These aspects that are usually dysfunctional might indeed increase performance in this specific task when is being carried out within the classroom. In the short time required to perform the task, within the classroom children were exposed to very few or any types of distractions. In addition, the task was presented as a game that somehow, due to social competition, forced the students’ focus on the task. 

Hence, while in the emotional Stroop task children exhibiting lower HRV were slower in shifting their attention during tasks with emotionally negative stimuli compared with positive ones, here where no significant distractors were present, a more “rigid” and systematic response to the task (lower HRV) was beneficial. In the first step of this work, in line with findings in previous similar tasks, students with higher HRV demonstrated similar reactions to negative emotional and positive stimuli, suggesting that they were more effective in managing their attention [27]. However, the opposite happened in a highly controlled environment (the classroom) when children performed a fairly new and short task that required a methodical approach to be solved [24]. In this case, those with lower HRV performed better. Empirical data not always report a positive relation between resting state HRV and attentional performance [40]. Findings might vary as a function of the task and its length [24]. The detection of as many cues as possible required in the Bells task might get advantage from a rigid way to scan the environment that is typical of low HRV. 

Moreover, HRV may be important in relation to the length of a task as in longer tasks attention maintenance gets harder and inhibitory and self-regulatory abilities become more important in order to succeed [24]. Much more data are needed here linking HRV at rest with this kind of task in children, also increasing the assessment time. Last, from this interaction it is clear how, in a green environment, individual difference in physiological self-regulation does not play a moderating role in sustained attention as nature in itself maintains attentional resources with less fatigue [13,39]. 

The study documents the positive effects of depicted and real green environments on young students’ control of attention and emotion. Methodological convergence of different tasks on these effects corroborates and strengthens the outcomes. The study theoretically contributes to the literature first by adding that the attentional benefits of a short exposure to nature in primary-school children emerge not only after a green break as documented in the literature [15], but also during the execution of selective and sustained attention tasks, which are greater in the greenness. Even when a green environment is only depicted in graphical stimuli, it distracts less from the emotionally negative ones. Second, the study adds to the current literature that a physiological correlate of attentional processes, such as self-regulation as indexed by heart rate variability, plays an important role in the relationship between indoor/outdoor environment and attentional performance. Specifically, physiological self-regulation consistently reveals to be more needed in relation to the classroom than the green environment. 

From a more practical point of view, it is noteworthy that today children spend less and less time outdoor and many have very little contact with nature. It is therefore important to give them the opportunities to have experiences with nature to support their attentional processes, which are fundamental for academic performance [10,41]. 

Our study also suggests that the use of some stimuli of a green environment may buffer, to some extent, the negative consequences for attention of third and fourth graders’ interaction with emotionally disturbing events that occur in the school setting. Moreover, interestingly, the study indicates that when primary-school children are involved in sustained and prolonged attentional processes in a green space, differences in their ability to cope with environmental requests at a physiological level do not play a role. This relevant and novel outcome implies that especially for tasks and activities requiring more on-going self-regulation than others during their execution, which is basically physiological, an outdoor school environment minimizes individual differences that could disadvantage some students from the first years of education [42]. 

Giving children systematic opportunities to spend time in green spaces for cognitive and emotional benefits [43] may also promote, in the long run, connectedness with nature and pro-environment attitude and behavior. Children who feel more connected to nature are also more inclined to sustainable behaviors, including more ecological actions [44]. 

Last but not least, it is practically important that the cognitive and emotional benefits of natural environments are relatively low cost and can be easily promoted by exposing children to greenness during school activities. An indirect way to contribute to young students’ greater academic and emotional functioning is to consider the relevance of the environment where learning activities take place: a green environment may make a difference. 

### Limitations

Like any study, the present too is not free from limitations. First of all, due to COVID-19 restrictions for collaborations with schools, the sample size is limited. Second, given the sample size, we could not control for a number of potentially relevant covariates, including abilities in basic cognitive processes and the perception of the classroom environment [29]. More solid results will be obtained in studies based on larger samples and that keep under control potentially individual differences that, beyond age, can play a role when investigating the effects of indoor/outdoor environment on control of attention and emotion. Third, we used a lab task to measure emotional attention and a typical task to measure selective and sustained attention. Future research can shed more light on the benefits of exposure to nature by also including tasks characterized by more ecological validity, such as assignments that are usually executed at school. We are moving in this direction. 

## 5. Conclusions

Despite these limitations, the study contributes to research on the positive effects of the natural environment on children’s control of attention and emotion. Specifically, findings reveal that third and fourth graders are less distracted from negative emotional materials when presented with green outdoor background stimuli than with indoor classroom backgrounds. Additionally, when completing a typical attentional task, children showed greater selective and sustained attention in the outdoor green environment than in the indoor classroom environment. Furthermore, sustained attention varied in relation to physiological self-regulation but only when performing the attentional task inside the classroom. Young students can therefore benefit from exposure to nature during a school day when performing tasks that require goal-driven, voluntary selective and sustained attention.

## Figures and Tables

**Table 1:** Linear Regression for Children’s Emotional Interference.

	*B*	*SE*	*t*	*p*
Environmental background	37.59	15.03	2.46	0.02
HRV	−43.12	11.20	−4.82	0.01
Age	−31.36	15.45	−2.03	0.03
EB × HRV	−23.36	10.45	−1.79	0.04
*R* ^2^	0.12			

Note. Environmental background coded 1 = Outdoor green background; 2 = Indoor classroom background; HRV = heart rate variability.

**Table 2:** Linear Regressions Models for Selective Attention and Sustained Attention.

	*B*	*SE*	*t*	*p*
Selective Attention				
Environment (outdoor vs. indoor)	−5.45	2.43	−2.24	0.02
HRV	−0.02	0.02	−1.75	0.25
Age	2.08	1.43	1.45	0.15
Environment × HRV	−0.00	0.03	−0.02	0.64
*R* ^2^	0.19			
Sustained Attention				
Environment (outdoor vs. indoor)	−4.83	1.07	1.45	0.05
HRV	−1.23	8.49	−0.00	0.10
Age	1.67	5.95	2.81	0.01
Environment × HRV	−2.53	1.25	−2.02	0.03
*R* ^2^	0.26			

Note. HRV = heart rate variability.

## Data Availability

The dataset generated during and/or analyzed during the current study is available from the corresponding author upon reasonable request.

## Supplementary Materials

The following supporting information can be downloaded at: https://www.mdpi.com/article/10.3390/ijerph192013141/s1. Figure S1: Study Design. Figure S2: Simple slopes for the interaction between environment (outdoor vs. classroom) and HRV on children’s emotional interference.
