# Supplementary material for: Children’s Attentional Processes in Outdoor and Indoor Environments: The Role of Physiological Self-Regulation
Source: Int J Environ Res Public Health. 2022 Oct 12;19(20):13141. doi: 10.3390/ijerph192013141 (PMC9602797; doi:10.3390/ijerph192013141)

Supplementary Material

Figure S1. Study Design

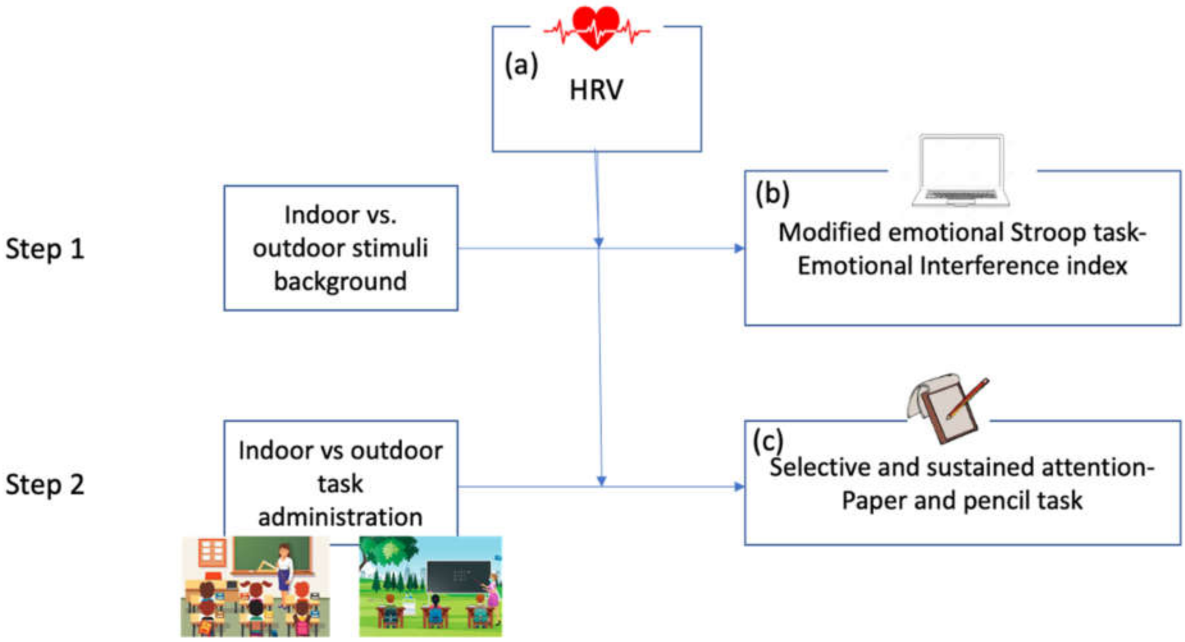

Note. All students were involved in both Steps 1 and 2. First, children were individually assessed to register (a) heart rate variability at rest. In the same individual session children's (b) reaction times were recorded in a modified version of the emotional Stroop task (Step 1). Last, children (c) were collectively assessed (as a class) on a paper and pencil attention task twice (inside the classroom and outside in the greenness (Step 2).

Figure S2. Simple Slopes for the Interaction between Environment (Outdoor vs. Classroom) and HRV on Students' Emotional Interference

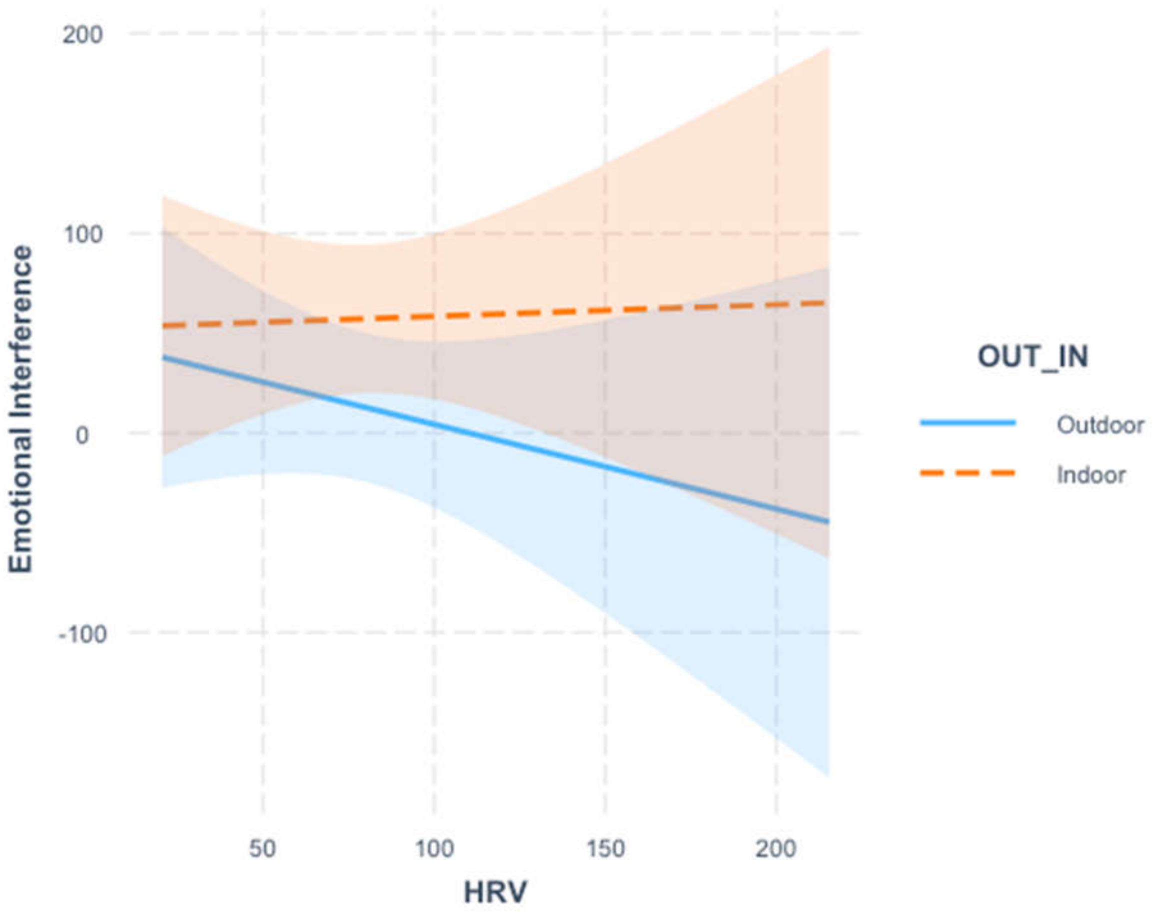

Supplement: Supplementary file 1 [file ijerph-19-13141-s001.zip › ijerph-1961750-supplementary.pdf]
